# Vascular endothelial cell-secreted exosomes facilitate osteoarthritis pathogenesis by promoting chondrocyte apoptosis

**DOI:** 10.18632/aging.202506

**Published:** 2021-02-01

**Authors:** Run-Ze Yang, Huo-Liang Zheng, Wen-Ning Xu, Xin-Feng Zheng, Bo Li, Lei-Sheng Jiang, Sheng-Dan Jiang

**Affiliations:** 1 Department of Clinic of Spine Center, Xinhua Hospital, Shanghai Jiao Tong University School of Medicine, Shanghai 200082, China

**Keywords:** ECs-Exo, apoptosis, anti-oxidative stress, osteoarthritis

## Abstract

Exosomes are major mediators of cell-to-cell communication, and are involved in many physiological and pathological processes. Recently, the roles of exosomes in osteoarthritis (OA) and their therapeutic potential have received increasing attention. Exosomes derived from vascular endothelial cells have been confirmed to participate in the occurrence and development of numerous diseases; however, their effects in OA have not been reported. Here, we demonstrated the roles of exosomes secreted by vascular endothelial cells in the development of OA. Through *in vivo* and *in vitro* experiments, we demonstrated that exosomes derived from vascular endothelial cells decreased the ability of chondrocytes to resist oxidative stress by inhibiting autophagy and p21 expression, thereby increasing the cellular ROS content and inducing apoptosis. These findings indicate that exosomes derived from vascular endothelial cells promote the progression of OA, thus, providing new ideas for the diagnosis and treatment of OA.

## INTRODUCTION

Osteoarthritis (OA) is one of the most common degenerative joint diseases worldwide, affecting more than 300 million people [1]. Mobility impairment and long-term chronic pain caused by OA severely decrease quality of life. In addition, patients with OA impose a substantial economic burden on society. Three main treatment methods for OA are currently used: nonpharmacological treatment, pharmacological treatment and surgical treatment [2–4]. Nonpharmacological treatments, for instance hot compresses, exercise and weight loss, are appropriate for patients with early-stage OA. The main objectives of drug therapy are to control pain and improve function and daily quality of life. Surgical treatment is widely used in end-stage patients with severe functional disability. At present, few satisfactory strategies are available to improve intra-articular homeostasis and delay the progression of OA [5]. Understanding the mechanisms underlying OA could aid in the development of new clinically needed therapies.

During the progression of arthritis, pathological changes in joints include cartilage damage, subchondral bone remodeling, synovial inflammatory activation, ligament and meniscus degeneration, and changes in the joint capsule, periarticular muscles, nerves and local fat pads. Although many factors have been found to be associated with the pathogenesis of OA, including aging, trauma, mechanical load, inflammation and metabolic disorders [4, 6], the exact etiology is unknown. One key factor known to lead to OA development is the production of high levels of inflammatory cytokines such as IL-1β, which contributes to disease progression [7]. Multiple studies have shown that IL-1β is involved in extracellular matrix degradation. In addition, IL-1β helps activate several chondrocyte proteolytic enzymes, including matrix metalloproteinases, and it additionally decreases proteoglycan and collagen synthesis [8]. Inflammatory activation of the synovium induces the release of different types of proinflammatory mediators, which not only cause extensive changes in synovial tissue structure and function, but also promote articular cartilage injury and accelerate the development of OA [9, 10].

Exosomes, membrane-bound vesicles approximately 40–150 nm in size, are produced by almost all cells, and modulate cell-cell communication by transferring biologically active molecules (lipids, nucleic acids and proteins) from source cells to target cells. Exosomes have been found to have anti-damage and pro-regenerative functions in various disease models, without causing clear adverse effects, such as immunogenicity or tumorgenicity [11–13]. Blood vessels play an important role in the skeletal system [14–16]. The growth of vascular networks in the skeletal system is controlled by osteoclasts, osteoblasts and other bone cells [17, 18]. Effective communication among these cells is essential to maintain bone homeostasis, and exosomes are important nanocarriers that transmit genetic information in this milieu. In recent years, the roles of vascular vessels in bone formation have received increasing attention [14, 15]. However, the effects of exosomes secreted by vascular endothelial cells (ECs) in OA have not been reported. In this study, we explored whether vascular EC-secreted exosomes (EC-Exos) might affect the progression of OA.

## RESULTS

### Isolation, identification and internalization of EC-Exos

**Figure 1:**
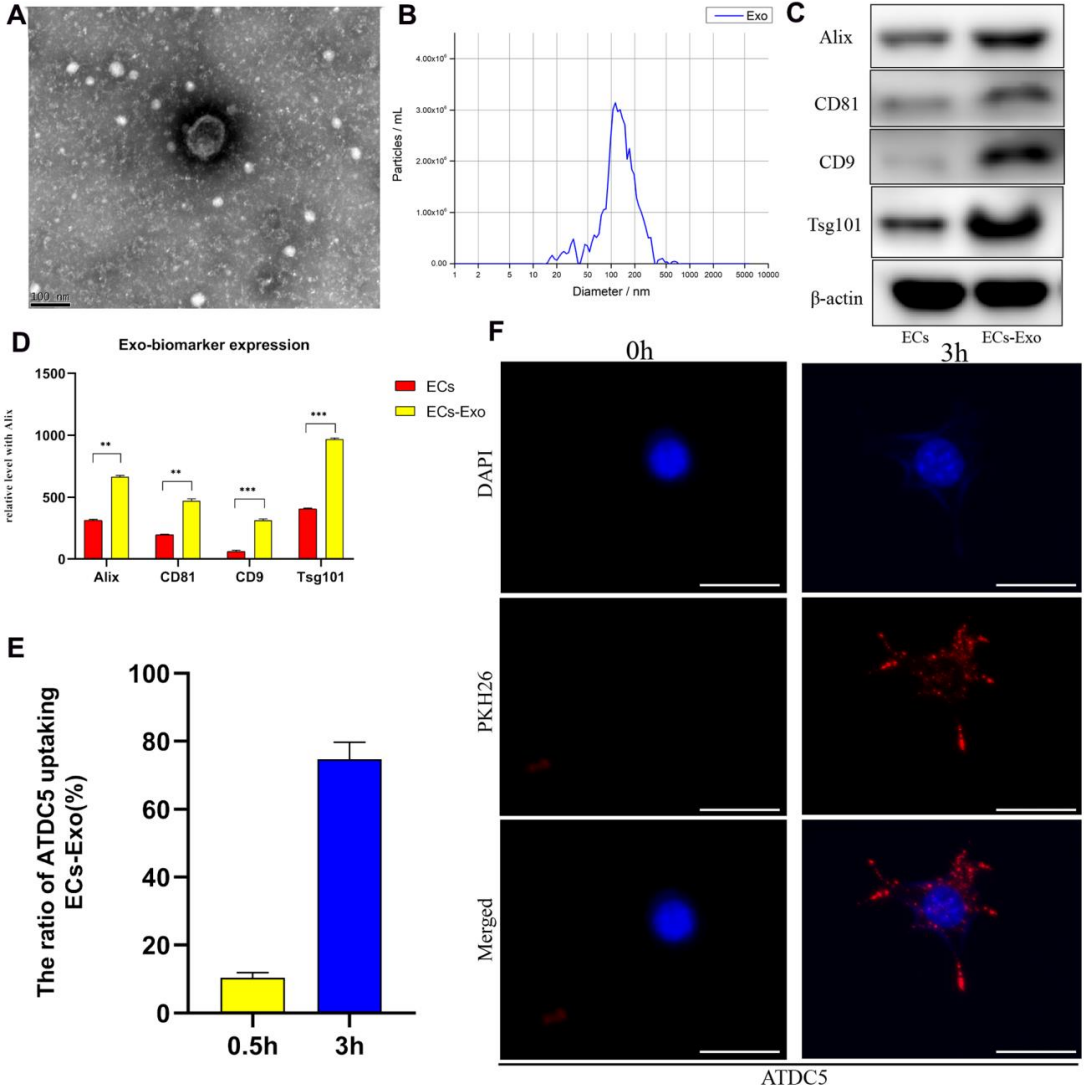
**Identification and internalization of EC-Exos.** Exosomes were isolated from samples with a total exosome extraction kit or ultrafiltration from mouse vascular EC medium. (**A**) Morphological features of EC-Exos were observed via transmission electron microscopy. (**B**) Particle sizes of exosomes were monitored with NTA. The X-axis shows the particle sizes in the sample, and the Y-axis shows the concentrations of particles of a certain size. Total protein was extracted from exosomes and analyzed with western blotting. (**C**, **D**) Representative images showing the expression of the exosome surface markers Alix, CD81, CD9 and Tsg101. PKH26-labeled exosomes were co-cultured with ATDC5 cells for 3 hours. (**E**) Quantitative analysis of the uptake rates of exosomes in ATDC5. (**F**) Immunofluorescence images showing the uptake of EC-Exos by ATDC5. The nuclei were labeled with DAPI (blue), and PKH26-labeled exosomes were internalized by ATDC5 cells (red). ***p<0.001, **p<0.01, *p<0.05 (n = 3).

We used a total exosome extraction kit or ultrafiltration to isolate mouse vascular EC-derived EC-Exos. The EC-Exos were identified on the basis of surface markers, particle size and morphological characteristics. First, transmission electron microscopy was used to detect the morphological features of isolated EC-Exos, and the observed 60–110 nm bilayer disc-like vesicles had characteristics consistent with exosomes (Figure 1A). Second, nanoparticle tracking analysis (NTA) was used to examine the exosome particle diameters and concentrations. As shown in Figure 1B, the particle sizes were primarily 90–180 nm, thus, implying that these exosomes were of high quality. To further confirm the isolation of extracellular vesicles, we performed western blotting for typical exosome biomarkers, such as Alix, CD81, CD9 and Tsg101. As shown in Figure 1C, 1D, under equal loading conditions, the quantity of Alix, CD81, CD9 and Tsg101 was clearly higher in EC-Exos than in the EC control. Because the absorption of exosomes into mouse chondrocytes was necessary in the early stages of the next experiment, we detected the uptake capacity of EC-Exos into mouse chondrocytes through fluorescence microscopy, after incubation with extracellular vesicles labeled with PKH26. After 3 hours, the exosome uptake rate of ATDC5 cells reached approximately 75%, and PKH26-labelled fluorescent spots appeared inside the mouse chondrocytes, thus, indicating that chondrocytes effectively internalized the EC-Exos (Figure 1E, 1F).

### EC-Exos attenuated antioxidant stress in mouse chondrocytes exposed to inflammation and increased the numbers of apoptotic cells

**Figure 2:**
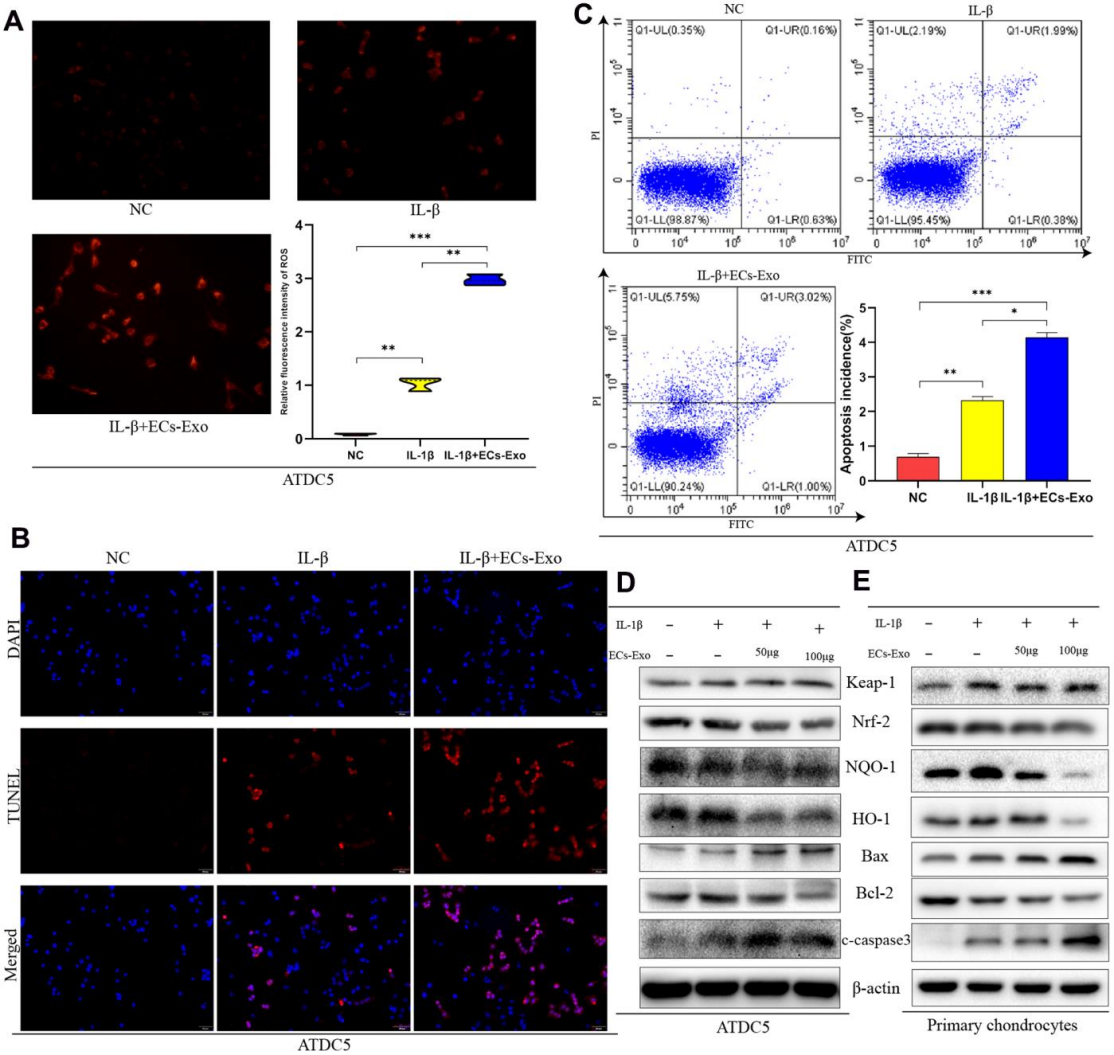
**EC-Exos decreased the anti-oxidative stress response of mouse chondrocytes stimulated with inflammation and increased the ratio of apoptotic cells.** ATDC5 cells and primary chondrocytes were exposed to IL-1β or IL-1β and EC-Exos at different concentrations (50 and 100 μg). (**A**) Comparison of ROS content in ATDC5 cells after specific experimental treatments. (**B**) Apoptosis was detected with TUNEL assays in ATDC5 cells under different treatments. (**C**) Flow cytometry was used to monitor apoptosis in dyed ATDC5 cells under different treatments. (**D**, **E**) Protein level of Keap-1, Nrf-2, NQO-1, HO-1, Bax, Bcl-2, C-caspase3 and β-actin in ATDC5 cells and primary chondrocytes under different treatments. ***p<0.001, **p<0.01, *p<0.05 (n = 3).

To simulate the inflammatory stimulation in OA, we treated ATDC5 cells and mouse primary chondrocytes with IL-1β. To study the effects of EC-Exos on OA development, we stimulated ATDC5 cells and mouse primary chondrocytes with 10 ng/ml IL-1β ± 50 μg or 100 μg EC-Exos for 24 h. Active oxygen detection experiments indicated that the content of ROS of ATDC5 cells exposed to IL-1β was higher than that in the NC cells, and EC-Exos further increased the levels of ROS beyond those in ATDC5 cells exposed to IL-1β (Figure 2A). TUNEL assays were used to monitor ATDC5 apoptosis. As shown in Figure 2B, the rate of apoptosis in ATDC5 cells increased after exposure to IL-1β and further increased in the presence of both IL-1β and EC-Exos. The results of Annexin V-FITC apoptosis detection also demonstrated that EC-Exos aggravated apoptosis in mouse chondrogenic cells stimulated with inflammation (Figure 2C). In western blotting assays, we found that the amount of C-caspase3 is increased, the expression levels of Keap-1and Bax were upregulated, and those of Nrf-2, HO-1, NQO-1 and Bcl-2 were downregulated in ATDC5 cells and primary chondrocytes exposed to IL-1β, thus, suggesting that inflammatory stimulation decreased the ability of mouse chondrocytes to resist oxidative stress and increased the incidence of apoptosis. As the concentration of EC-Exos increased, the protein expression of Nrf-2, HO-1, NQO-1 and Bcl-2 was further downregulated in ATDC5 cells and primary chondrocytes, thus indicating that exosomes derived from vascular ECs further weakened the anti-oxidative stress response of mouse chondrocytes under inflammatory stimulation and increased apoptosis (Figure 2D, 2E). The ratios of apoptosis marker proteins to β-actin and anti-oxidative stress associated proteins to β-actin further indicated that after IL-1β stimulation, the cells’ ability to resist oxidative stress decreased, and the rate of apoptosis increased. However, the resistance to oxidative stress further declined, and the rate of cell apoptosis further increased in both ATDC5 cells and primary chondrocytes exposed to IL-1β and EC-Exos (Supplementary Figure 1A–1L).

### Enhanced autophagy decreased apoptosis in ATDC5 cells exposed to IL-1β and EC-Exos and increased their ability to resist oxidative stress

**Figure 3:**
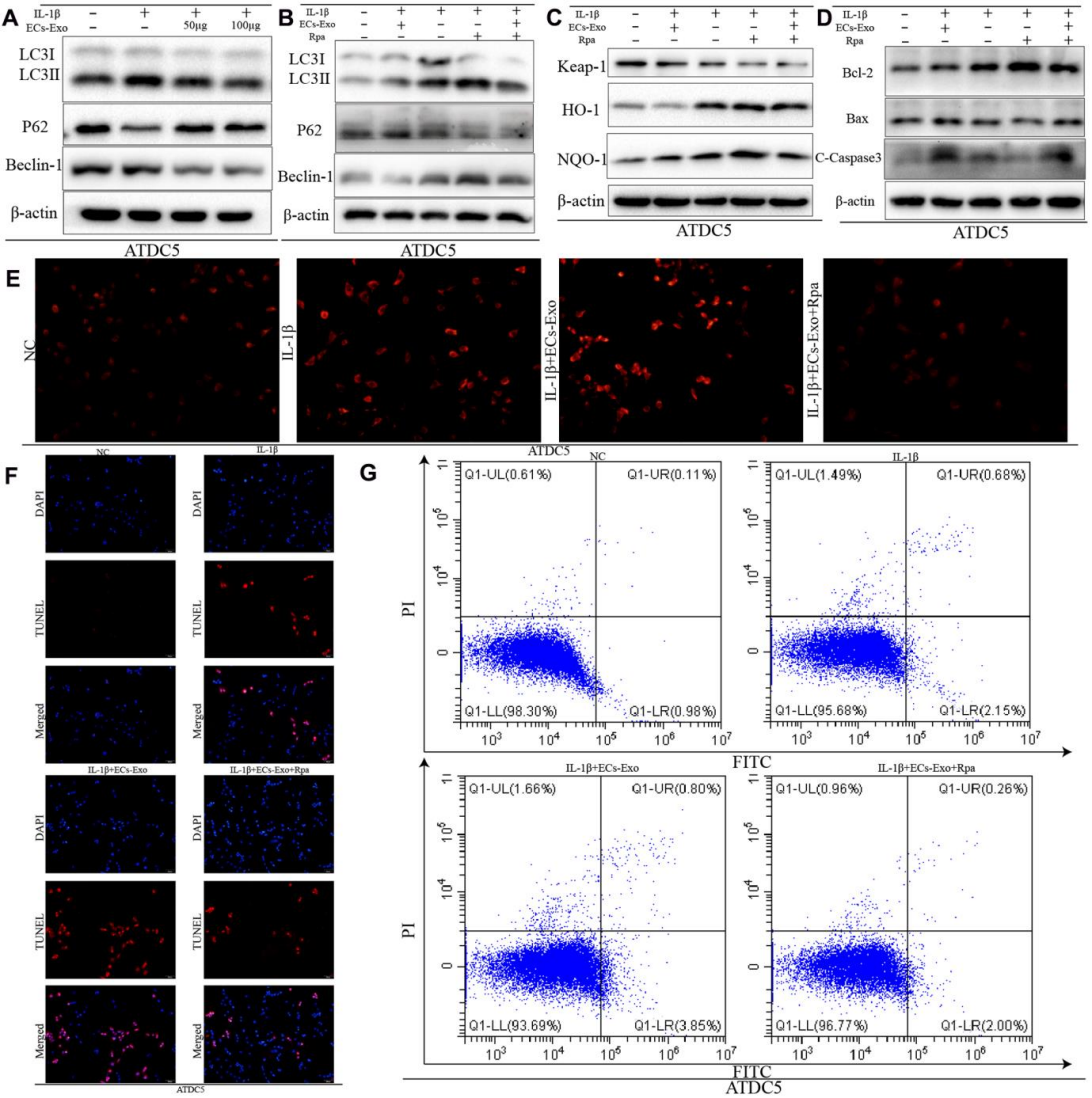
**Increased autophagy decreased the apoptosis and intracellular ROS content in ATDC5 cells treated with IL-1β and EC-Exos.** ATDC5 cells were treated with 10 ng/ml IL-1β ± EC-Exos (100 μg) or rapamycin (50 nmol/L) for 24 h. (**A**–**D**) Protein level of LC3I, LC3II, P62, Beclin-1, Keap-1, HO-1, NQO-1, Bcl-2, Bax, C-caspase3 and β-actin in ATDC5 cells under different treatments. (**E**) ROS content in ATDC5 cells after different specific experimental treatments. (**F**) TUNEL assays were used to monitor apoptosis in ATDC5 cells treated with different methods. (**G**) Apoptosis of ATDC5 cells under different treatments, as detected by flow cytometry.

We found that the expression of LC3II and Beclin-1 increased, and the expression of P62 decreased, in ATDC5 cells exposed to IL-1β, in contrast to the control cells. However, in ATDC5 cells exposed to IL-1β, incubation with EC-Exos resulted in downregulated LC3II and Beclin-1 expression, and upregulated P62 expression, as compared with that in ATDC5 cells stimulated with only IL-1β (Figure 3A). These results implied that autophagy increased in ATDC5 cells under inflammatory stress, but this increase was reversed in ATDC5 cells incubated with EC-Exos and IL-1β. The ratios of LC3II/I, p62/β-actin and Beclin-1/β-actin also demonstrated that EC-Exos reversed the autophagy in ATDC5 cells treated with IL-1β (Supplementary Figure 1M, 1N). Rapamycin was used to activate autophagy; as shown in Figure 3B and Supplementary Figure 2A, 2B, rapamycin successfully upregulated autophagy regardless of whether the cells stimulated with IL-1β were treated with EC-Exos. To further explore the effects of autophagy on apoptosis and oxidative stress after activation, we performed western blot assays. As shown in Figure 3C, 3D, the expression of Keap-1and Bax decreased, the amount of C-caspase3 is decreased and that of HO-1, NQO-1 and Bcl-2 increased in ATDC5 cells incubated with IL-1β, EC-Exos and rapamycin, as compared with ATDC5 cells exposed to IL-1β and EC-Exos, thus indicating that increased autophagy increased the cells’ ability to resist oxidative stress and reversed the increase in apoptosis in ATDC5 cells stimulated with IL-1β and EC-Exos. The ratios of apoptosis marker proteins to β-actin and anti-oxidative stress associated proteins to β-actin also illustrated that the anti-oxidative stress response increased, and the cell apoptosis declined in ATDC5 cells treated with IL-1β, EC-Exos and rapamycin, as compared with cells exposed to IL-1β and EC-Exos (Supplementary Figure 2C, 2D). ROS measurements indicated that the levels of ROS in ATDC5 cells exposed to IL-1β, EC-Exos and rapamycin were lower than those in ATDC5 cells exposed to IL-1β and EC-Exos (Figure 3E). Figure 3F, 3G also show that the increase in autophagy was associated with a decreased ratio of apoptotic ATDC5 cells stimulated with IL-1β and EC-Exos.

### Overexpression of p21 enhanced the ability to resist oxidative stress and decreased apoptosis in ATDC5 cells exposed to IL-1β and EC-Exos

**Figure 4:**
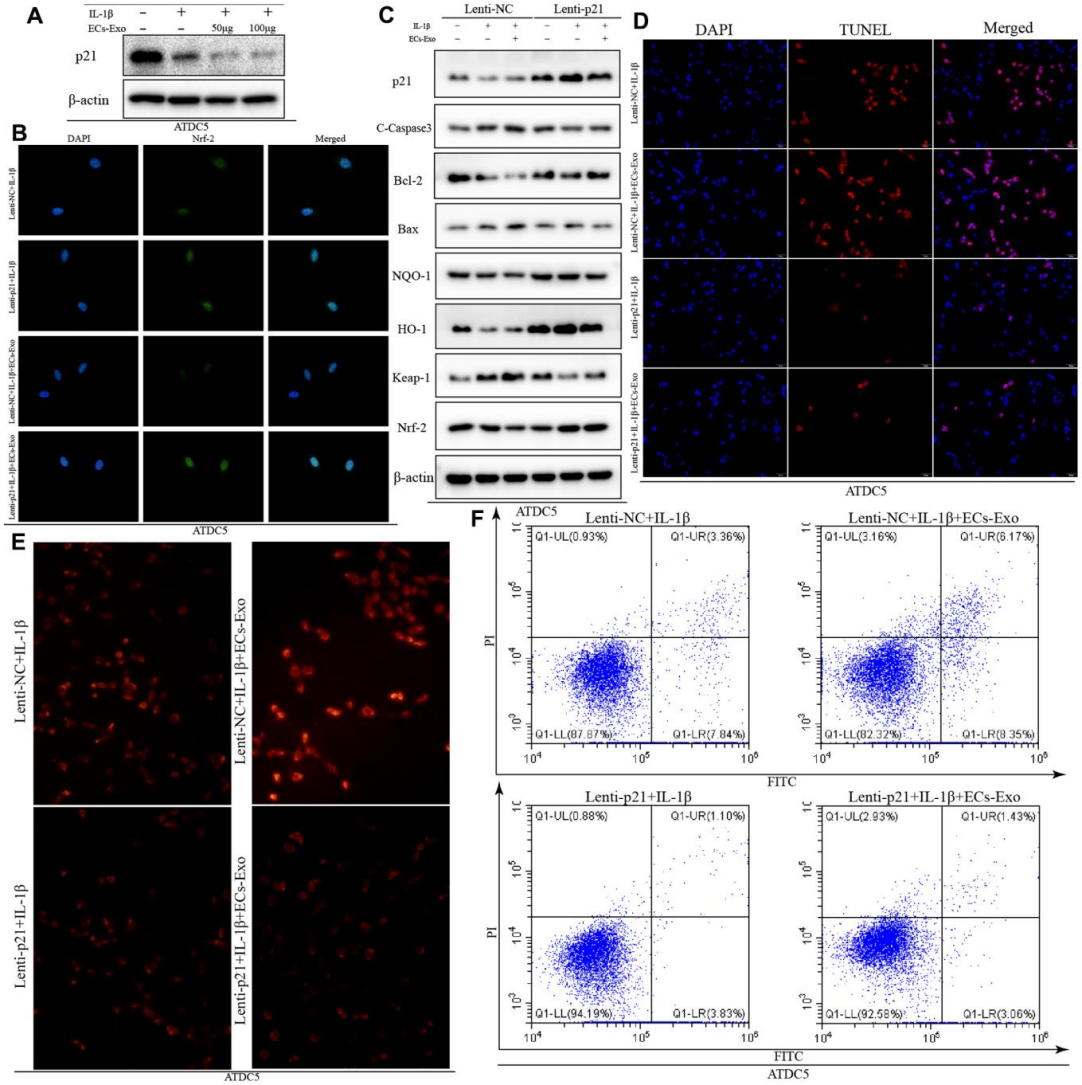
**Overexpression of p21 increased the anti-oxidative stress response and decreased the ratio of apoptotic ATDC5 cells exposed to IL-1β and EC-Exos.** ATDC5 cells transfected with Lenti-NC or Lenti-p21 were exposed to 10 ng/ml IL-1β ± EC-Exos (100 μg) for 24 h. (**A**) Protein expression of p21 and β-actin in ATDC5 cells treated with IL-1β or IL-1β and EC-Exos at different concentrations (50 and 100 μg). (**B**) Immunofluorescence assay of Nrf-2 in ATDC5 cells under different treatments. (**C**) Protein level of p21, C-caspase3, Bcl-2, Bax, NQO-1, HO-1, Keap-1, Nrf-2 and β-actin in ATDC5 cells treated with various methods. (**D**) TUNEL assays were used to detect apoptosis in ATDC5 cells under different treatments. (**E**) ROS content in ATDC5 cells under different treatments (**F**) Flow cytometry was used to detect apoptosis in dyed ATDC5 cells exposed to different experimental treatments.

On the basis of recent reports, p21, a multifunctional molecule, promotes activation of the nuclear factor E2-related factor 2 (Nrf2)/heme oxygenase-1 (HO-1) pathway, which plays an important role in the regulation of apoptosis induced by oxidative stress [19, 20]. Therefore, we detected changes in p21 expression in ATDC5 cells under different treatment conditions. As shown in Figure 4A, the expression of p21 in ATDC5 cells exposed to IL-1β was lower than that in ATDC5 cells. As the concentration of added EC-Exos increased, the expression of p21 continued to decline in ATDC5 cells exposed to IL-1β and EC-Exos. The ratio of p21 to β-actin also indicated that the expression of p21 was downregulated in ATDC5 cells exposed to IL-1β compared with control cells, and this decrease was aggravated in ATDC5 cells incubated with IL-1β and EC-Exos (Supplementary Figure 2E). Lentivirus transfection was used to overexpress p21; as shown in Figure 4C, the expression of p21 in ATDC5 cells was clearly higher in the Lenti-p21 group than the Lenti-NC group. Western blotting indicated that the expression of Bcl-2, NQO-1, HO-1 and Nrf-2 was upregulated in ATDC5 cells treated with IL-1β or IL-1β and EC-Exos in the Lenti-p21 group, as compared with the Lenti-NC group. Moreover, the amount of C-caspase3, Bax and Keap-1 was downregulated in ATDC5 cells exposed to IL-1β or IL-1β and EC-Exos in the Lenti-p21 group, in contrast to the Lenti-NC group (Figure 4C). The ratios of anti-oxidative stress associated proteins to β-actin, and apoptosis marker proteins to β-actin also demonstrated that overexpression of p21 reversed the decline in cellular antioxidant stress induced by inflammatory stimulation and EC-Exos, and decreased the occurrence of apoptosis (Supplementary Figure 2F–2K). Immunofluorescence detection was used to monitor the expression of Nrf-2. As shown in Figure 4B, the green fluorescence intensity of Nrf-2 in the ATDC5 cells in the Lenti-P21 group was stronger than that in the Lenti-NC group. The ROS levels were also consistent with the results of western blotting. The content of ROS in ATDC5 cells in the Lenti-NC and IL-1β group was greater than that in the Lenti-p21 and IL-1β group, and that in the Lenti-NC, IL-1β and EC-Exo group was greater than that in the Lenti-p21, IL-1β and EC-Exo group (Figure 4E). TUNEL assay and Annexin V-FITC apoptosis detection indicated that overexpression of p21 decreased the incidence of apoptosis in ATDC5 cells treated with IL-1β or IL-1β and EC-Exos (Figure 4D, 4F).

### Exosomes derived from vascular ECs exacerbated degeneration of the knee joint in osteoarthritis model mice

**Figure 5:**
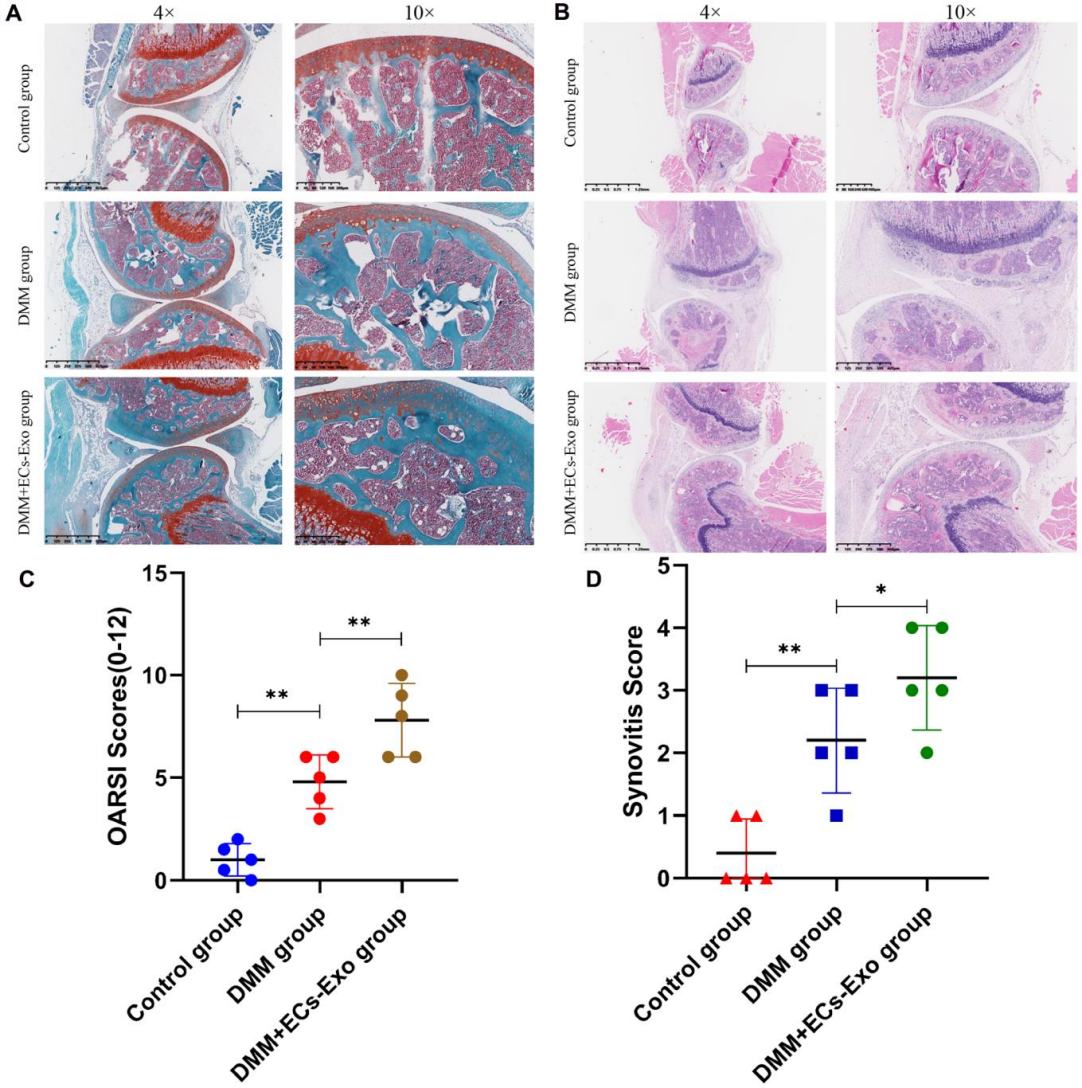
**EC-Exos promoted the progression of OA *in vivo*.** (**A**, **B**) Representative S-O staining and HE staining of knee-joint samples. (**C**) OARSI scores of knee joints in three groups of mice. (**D**) Synovitis scores of knee joints in three groups of mice. ***p<0.001, **p<0.01, *p<0.05 (n = 5).

To study the effects of exosomes secreted by vascular ECs on OA *in vivo*, we constructed a mouse model of osteoarthritis and performed EC-Exo treatment. Safranin O and Fast Green staining and hematoxylin and eosin staining were used to evaluate the progress of OA. The results illustrated that the knee joints of mice degenerated after surgery for destabilization of the medial meniscus (DMM), as compared with the control treatment; moreover, EC-Exos accelerated the progression of OA and increased the knee joint degeneration in the DMM+EC-Exo group compared with the DMM group (Figure 5A, 5B). The OARSI score and synovitis score in the control group, DMM group and DMM+EC-Exo group also demonstrated that EC-Exos exacerbated the progression of OA *in vivo* (Figure 5C, 5D).

### EC-Exos decreased chondrocyte resistance to oxidative stress and increased the number of apoptotic chondrocytes in the OA mouse model

**Figure 6:**
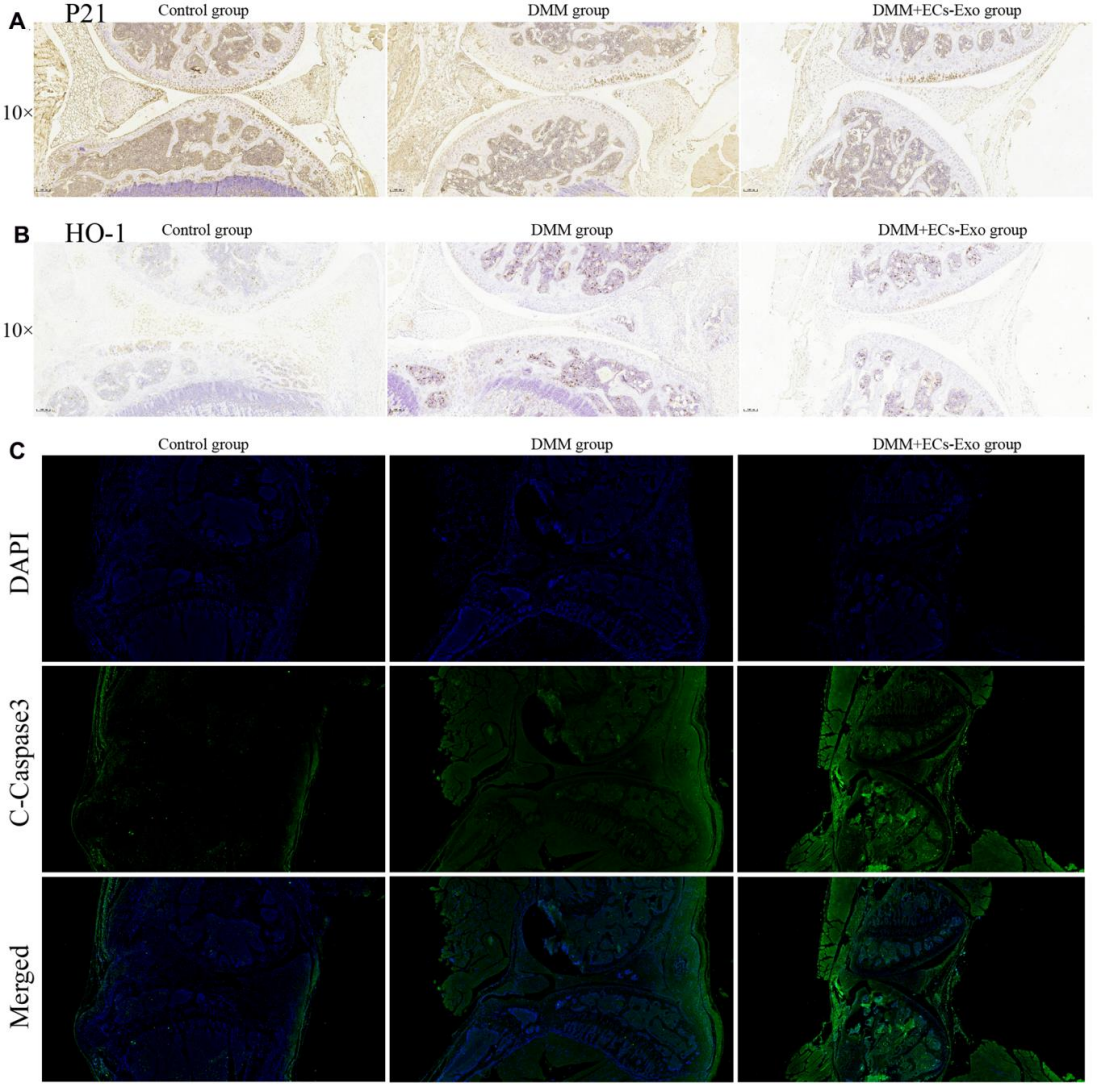
**EC-Exos decreased the ability of chondrocytes to resist oxidative stress and increased the number of apoptotic chondrocytes in the OA mouse model.** (**A**) Immunohistochemical staining for p21 expression in the knee-joint samples. (**B**) Immunohistochemical staining for HO-1 expression in the knee-joint samples. (**C**) Immunofluorescence staining for C-caspase3 expression in the knee-joint specimens from three groups of mice.

To further demonstrate the effects of EC-Exos on oxidative stress and apoptosis in chondrocytes during OA *in vivo*, we performed immunohistochemistry and immunofluorescence experiments. As shown in Figure 6A, 6B, among the three groups, the expression levels of p21 and HO-1 in the DMM+EC-Exo group were lowest, followed by those in the DMM group. However, the amount of C-caspase3 in the DMM+EC-Exo group was the highest, followed by that in the DMM group, among the three groups (Figure 6C).

## DISCUSSION

Many studies have demonstrated that exosomes play important roles in the occurrence and development of OA [21–24]. However, the effects of secreted exosomes on OA in the most widely distributed vascular ECs in the human body have not been reported. In this study, we demonstrated that EC-Exos promoted the occurrence and development of OA. First, mouse vascular EC-derived exosomes were isolated separately by density gradient centrifugation and an exosome extraction kit, and were analyzed by transmission electron microscopy, NTA and immunoblotting of surface markers, such as Alix, CD81, CD9 and Tsg101. PKH26 staining demonstrated that EC-Exos were phagocytosed by ATDC5 cells.

Through *in vitro* experiments, we found that EC-Exos increased the expression of Keap-1 and decreased the expression of Nrf-2, NQO-1 and HO-1 in ATDC5 cells and mouse primary chondrocytes exposed to IL-1β. These results implied that EC-Exos decreased the anti-oxidative stress response in ATDC5 cells and mouse primary chondrocytes stimulated with IL-1β. ROS measurements further supported this conclusion. Studies have shown that oxidative stress is closely associated with apoptosis, and if the ability of cells to resist oxidative stress is diminished, intracellular ROS increase and lead to cell apoptosis [25–28]. Western blotting was used to detect the expression of apoptosis-associated proteins. The results of flow cytometry analysis of apoptosis and TUNEL assays were consistent with the results of western blotting. Our findings together demonstrated that EC-Exos further promoted apoptosis in ATDC5 cells exposed to IL-1β.

Detection of autophagy associated protein expression indicated that IL-1β upregulated autophagy in ATDC5 cells, whereas EC-Exos reversed this increase and downregulated autophagy in ATDC5 cells exposed to IL-1β. As also shown in previous studies, autophagy acts as a double-edged sword: moderate autophagy can eliminate harmful substances in cells and decrease apoptosis, whereas excessive autophagy can induce the production of harmful substances such as ROS, thereby inducing apoptosis [29–31]. We presumed that IL-1β would induce protective autophagy, but EC-Exos might restrict this autophagy in ATDC5 cells. To verify this hypothesis, we used rapamycin to enhance autophagy. After this treatment, we found that the intracellular ROS content and the incidence of apoptosis decreased in ATDC5 cells exposed to IL-1β, EC-Exos and rapamycin, in contrast to the results in ATDC5 cells exposed to IL-1β and EC-Exos. This finding was consistent with our hypothesis.

P21, a multifunctional cellular protein, plays a vital role in anti-oxidative stress through regulating the Keap1/Nrf2/HO-1 pathway [32–34]. The results of western blotting illustrated that the expression of p21 was clearly lower in ATDC5 cells treated with IL-1β and EC-Exos than in ATDC5 cells treated with only IL-1β. After overexpression of p21, the expression of C-caspase3, Bax and Keap-1 was lower, and the expression of Bcl-2, NQO-1, HO-1 and Nrf-2 was higher in ATDC5 cells treated with IL-1β or IL-1β and EC-Exos than in the ATDC5 cells in the Lenti-NC group. ROS and apoptosis detection also demonstrated that overexpression of p21 enhanced the anti-oxidative stress response and decreased apoptosis in ATDC5 cells exposed to IL-1β and EC-Exos.

Animal experiments showed that the knee joints in the DMM group mice, as compared with those in the control group, were clearly degenerated. Moreover, the degree of knee joint degeneration in the DMM+EC-Exo group mice was greater than that in the DMM group. The OARSI score and synovitis score further supported these findings. Immunohistochemistry and immunofluorescence analyses demonstrated that EC-Exos decreased resistance to oxidative stress and increased apoptosis in osteoarthritic mouse chondrocytes.

## CONCLUSIONS

The mechanisms underlying OA remain unknown and require further investigation. Our study demonstrated that EC-Exos decreased the ability of chondrocytes to resist oxidative stress by inhibiting autophagy and P21 expression, thereby increasing the content of cellular ROS and inducing apoptosis. This response aggravated the progression of OA. Although further research is necessary, inhibiting exosome secretion from vascular ECs in joints might serve as a potential therapeutic strategy for OA.

## MATERIALS AND METHODS

### Cell culture and treatment experiments

Mouse vascular ECs (bEND.3) and the murine chondrogenic cell line ATDC5 were purchased from the American Type Culture Collection (ATCC, USA). ATDC5 cells were cultured in Dulbecco's modified Eagle's medium DMEM/F12 (HyClone, USA) supplemented with 10% fetal bovine serum (FBS; Gibco, USA), 100 U/mL penicillin and 100 mg/mL streptomycin at 37° C under 5% CO_2_ humidified air. Mouse vascular ECs were cultured at 37° C with 5% CO_2_, in DMEM/HG (HyClone, USA) containing 10% FBS and 1% penicillin–streptomycin (Gibco, Thermo Fisher Scientific, USA). When 80% confluence was reached, cells were seeded into appropriate plates for subsequent analysis.

Primary chondrocytes were isolated from 5-day-old mouse knee cartilage. The knee joints, with adjacent muscles, bone tissues and ligaments removed, were first digested with 0.25% trypsin-EDTA (Thermo Fisher Scientific, USA) at 37° C for 15 min. Then 0.1% collagenase II (Gibco/Life Technologies, USA) was used overnight at 37° C in an additional digestion to obtain chondrocytes from cartilage [35]. Cells were seeded in six-well plates to continue cultivation in DMEM/F12 supplemented with 1% penicillin/streptomycin and 10% FBS under a 5% CO_2_ environment at 37° C.

For the treatment with the cytokine IL-1β, ATDC5 cells and mouse primary chondrocytes were treated with 10 ng/ml of recombinant human IL-1β (PeproTech, USA) for 24 h. For experiments with exosomes produced by EC treatment, cells were treated with 50 μg or 100 μg exosomes for 24 h. In experiments using the autophagy activator rapamycin (MedChemExpress, China), ATDC5 cells were treated with 50 nmol/L rapamycin for 24 h.

### Isolation and identification of exosomes

After the cell density had reached 80%, ECs were washed twice with PBS, and then the medium was replaced with serum-free DMEM/HG containing 1% penicillin/streptomycin. After 48 h, the conditioned medium of ECs was collected to obtain exosomes. We used two methods for isolation of exosomes in this study: Total Exosome Isolation Reagent (from cell culture media) (Invitrogen, Thermo Fisher, USA) and ultrafiltration. The features of exosomes extracted through the two methods did not significantly differ. Briefly, harvested culture EC media were centrifuged at 300 g for 10 min and then at 1500 g for 10 min at 4° C. After centrifugation, the supernatant was filtered with a 0.22-μm filter (Steritop, Millipore, USA), and the remaining cells and debris were clearly removed. In the first method, the total exosome isolation reagent was mixed into the medium and incubated overnight at 4° C. The mixture was then centrifuged at 10000 g for 60 min for pellet retrieval. In the second method, the supernatant was ultra-centrifuged (HITACHI, Japan) at 100000 g for 70 min. Before the next step, the pallet was washed with PBS to remove protein contamination and then centrifuged at 100000 g for another 70 min. Exosomes isolated with the different methods were stored at −80° C for further use. We used a BCA protein assay kit (Beyotime, China) to determine the protein concentrations in the exosome suspensions. NTA of exosomes was performed with ZetaView (Particle Metrix, Germany). Transmission electron microscopy was used to identify exosome morphology. Western blotting analysis was used to detect surface marker proteins, including CD81, CD9, Alix and Tsg101 in exosomes.

### Uptake of EC-Exos by ATDC5

For exosome internalization detection, EC-Exos were marked with PKH26 red fluorescent dye (Sigma, USA) according to the manufacturer’s protocol. Briefly, exosomes (100 μg) were suspended in 500 μl of diluent C. Dilute dye in equal amounts (5 μl of PKH26) was then added into the exosome suspension and incubated for 5 min at 37° C. The staining reaction was terminated with 250 μl of 5% bovine serum albumin (Beyotime, China), and the PKH26-labeled exosomes were then co-cultured with ATDC5 cells for 3 h. Ultimately, cells were fixed with 4% paraformaldehyde, and nuclei were stained with 4′, 6-diamidino-2-phenylindol (DAPI) (Beyotime, China). Images were obtained with a fluorescence microscope.

### Western blotting

The western blotting analysis was performed according to a protocol described previously [36]. In brief, proteins extracted with RIPA (Beyotime, China), including PMSF (Beyotime, China) were separated by SDS-PAGE and electroblotted onto PVDF membranes (Millipore). The membranes were then blocked with 5% nonfat milk for 2 h and incubated overnight at 4° C with primary antibodies against Keap-1 (1:1000, Proteintech, China), Nrf-2 (1:1000, Proteintech, China), HO-1 (1:1000, Proteintech, China), NQO-1 (1:1000, Proteintech, China), Bax (1:1000, Proteintech, China), Bcl-2 (1:1000, Proteintech, China), cleaved caspase-3 (1:1000, Cell Signaling Technology, USA), p21 (1:1000, Abcam, UK), p62 (1:1000, Abcam, UK), Beclin-1 (1:1000, Proteintech, China), LC-3 (1:1000, Novus, USA) and β-actin (1:1000, Proteintech, China). Afterward, the membranes were washed with TBST three times and incubated with the corresponding horseradish peroxidase-conjugated secondary antibodies at room temperature for 1 h. Finally, the membranes were detected with ECL plus reagent (Millipore) with a ChemiDoc™ XRS + System (Bio-Rad, USA).

### TUNEL assay

The apoptosis of cells was monitored with a one-step TUNEL apoptosis assay kit (Beyotime, China) according to the manufacturer’s protocol. Briefly, the cells were treated and subsequently fixed with 4% paraformaldehyde at room temperature for 20 min, then incubated with 0.1% Triton X-100 (Beyotime, China) for 8 min and washed three times in PBS for 5 min each. Afterward, the cells were incubated with 50 μl TUNEL reaction mixture for 1 h at 37° C in the dark. The nuclei were stained with DAPI. Finally, the expression of apoptotic cells was observed under a fluorescence microscope.

### Flow cytometry analysis of apoptosis

An Annexin V-FITC apoptosis detection kit (Beyotime, China) was used to detect cell apoptosis. After cell treatments, cell samples were collected from 6 cm culture plates, transferred to 1.5 ml centrifuge tubes and resuspended with 195 μl 1× binding buffer. Finally, 5 μl Annexin V-FITC and 10 μl PI were added to the tubes. After incubation for 20–30 min, the samples were immediately analyzed with flow cytometry.

### ROS measurement

A Reactive Oxygen Species Assay Kit (Beyotime, China) was used to detect the overall intracellular ROS levels according to the manufacturer’s protocol. ATDC5 cells were treated with different concentrations EC-Exos, 10 ng/ml IL-1β and 50 nmol/L rapamycin for 24 h, then washed with PBS three times and incubated with serum-free DMEM/HG containing DCFH-DA at 37° C for 30 min in the dark. The medium was then removed, and the cells were washed with PBS three times. Fluorescence microscopy analysis (Olympus, Fluoview, Japan) was used to determine the ATDC5 cells’ fluorescence distribution. Positive cells emitted red light.

### Lentivirus transfection

Lenti-NC or Lenti-P21 was added at 50%–70% cell density. After 24 h, the medium was removed and replaced with fresh medium. The efficiency of transfection was determined with western blotting.

### Immunofluorescence examination

Immunofluorescence staining was performed as previously described [37]. After removal of the medium and washing with PBS, treated ATDC5 cells were fixed with 4% paraformaldehyde for 20 min, then permeabilized the with 0.1% Triton X-100 for 10 and blocked with 5% bovine serum albumin for 1 h. Cell samples were then incubated with primary antibodies at 4° C overnight in a humidified atmosphere. The next morning, cells were washed and incubated with fluorescein isothiocyanate or tetramethyl rhodamine isothiocyanate-conjugated secondary antibodies for 1 h at 25° C and labeled with DAPI for 5 min. Fluorescence microscopy was used to observe the results.

### Surgical mouse model of OA

Fifteen 10-week-old C57BL/6 male mice were purchased from the Animal Center of the Chinese Academy of Sciences (Shanghai, China). Mouse experiments were conducted according to the International Guiding Principles for Biomedical Research Involving Animals and were approved by the Ethics Committee of Xinhua Hospital Affiliated with Shanghai Jiao Tong University School of Medicine. DMM surgery was performed on the mice according to the procedure he described in a previous study [38]. After anesthesia with 2% pentobarbital sodium (40 mg/kg), the joint capsule was incised, and then the medial meniscotibial ligament was transected with microsurgical scissors. The animals were randomly divided into three groups: a control group, DMM group and DMM+EC-Exo group. In the control group, sham operation was performed on the bilateral knee joints with medial capsulotomy only. In the DMM group, bilateral knee joint DMM surgery was performed. In the DMM+EC-Exo group, in addition to performing DMM surgery, we injected EC-Exos (100 μg each) through the tail vein once per week.

### Histopathologic analysis

The mice were sacrificed with an intraperitoneal injection of an overdose of 10% pentobarbital sodium 6 weeks after surgery. Safranin O, Fast Green staining and hematoxylin and eosin staining were used to observe morphological changes in the cartilage and the subchondral bone under a microscope. Immunofluorescence was used to measure C-caspase3 expression in the cartilage, and immunohistochemical analysis was used to determine the expression of p21 and HO-1.

### Statistical analysis

All experiments were performed at least three times. The results are expressed as mean ± SD. Statistical analyses were performed in SPSS statistical software 20.0. In addition, Student's t test was used to analyze significant differences between independent groups, and differences across multiple groups were compared with one-way ANOVA. *p < 0.5, **p < 0.01 and ***p < 0.001 were considered significant differences.

## Supplementary Material

Supplementary Figures
