# Supplementary material for: Vascular endothelial cell-secreted exosomes facilitate osteoarthritis pathogenesis by promoting chondrocyte apoptosis
Source: Aging (Albany NY). 2021 Feb 1;13(3):4647–62. doi: 10.18632/aging.202506 (PMC7906201; doi:10.18632/aging.202506)
Supplement: Supplementary Figures [file aging-13-202506-s001.pdf]

SUPPLEMENTARY FIGURES

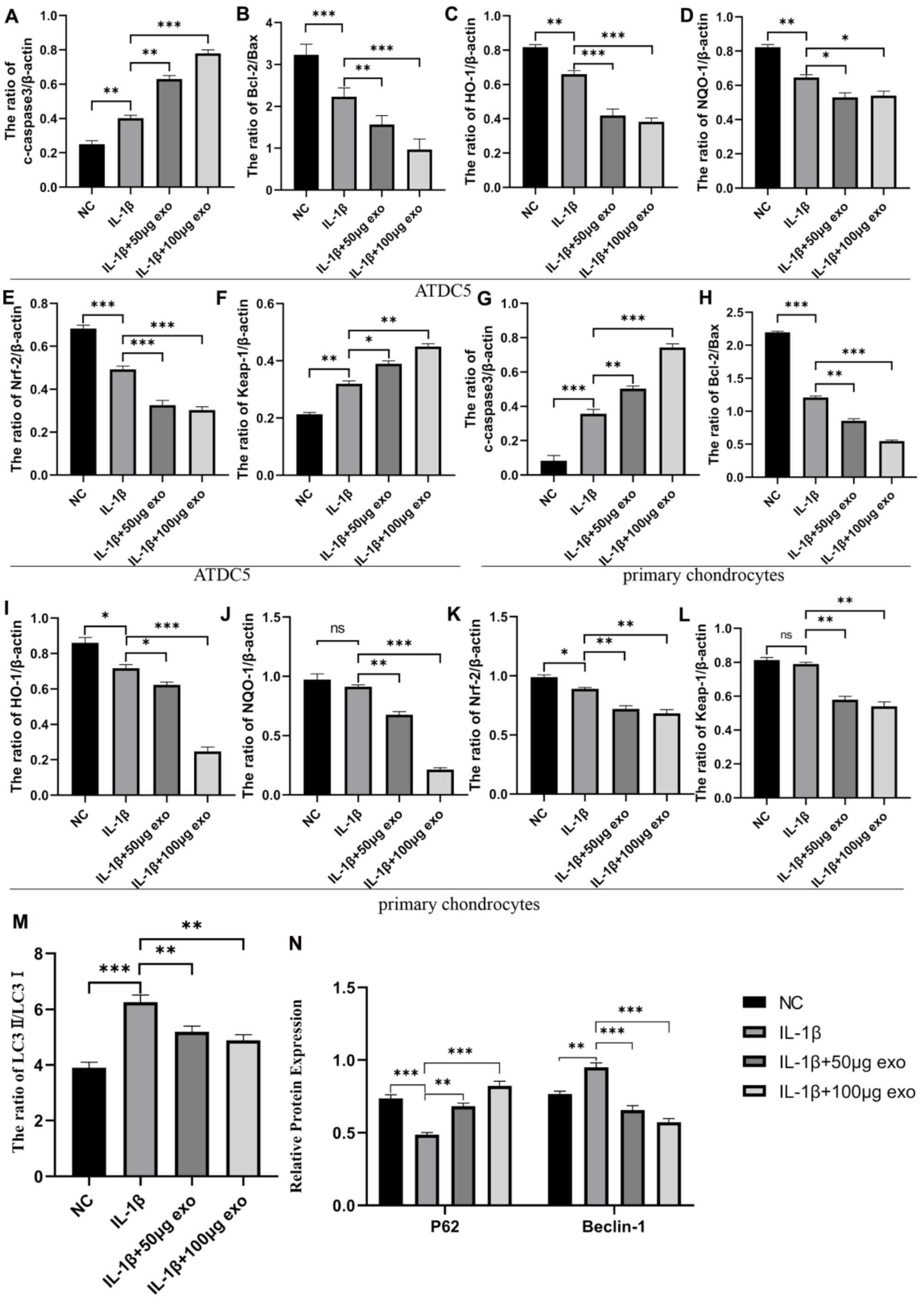

Supplementary Figure 1. (**A**–**F**) The ratio of c-caspase3/β–actin, Bcl-2/Bax, HO-1/β–actin, NQO-1/β–actin, Nrf-2/β–actin and Keap-1/β–actin in ATDC5 cells under different treatments. (**G**–**L**) The ratio of c-caspase3/β–actin, Bcl-2/Bax, HO-1/β–actin, NQO-1/β–actin, Nrf-2/β–actin and Keap-1/β–actin in primary chondrocytes under different treatments. (**M**) The ratio of LC3II/LC3I in ATDC5 cells in different groups. (**N**) The ratio of autophagy marker proteins (p62 and Beclin-1) to β–actin in ATDC5 cells in different groups. ***p<0.001, **p<0.01, *p<0.05 (n = 3).

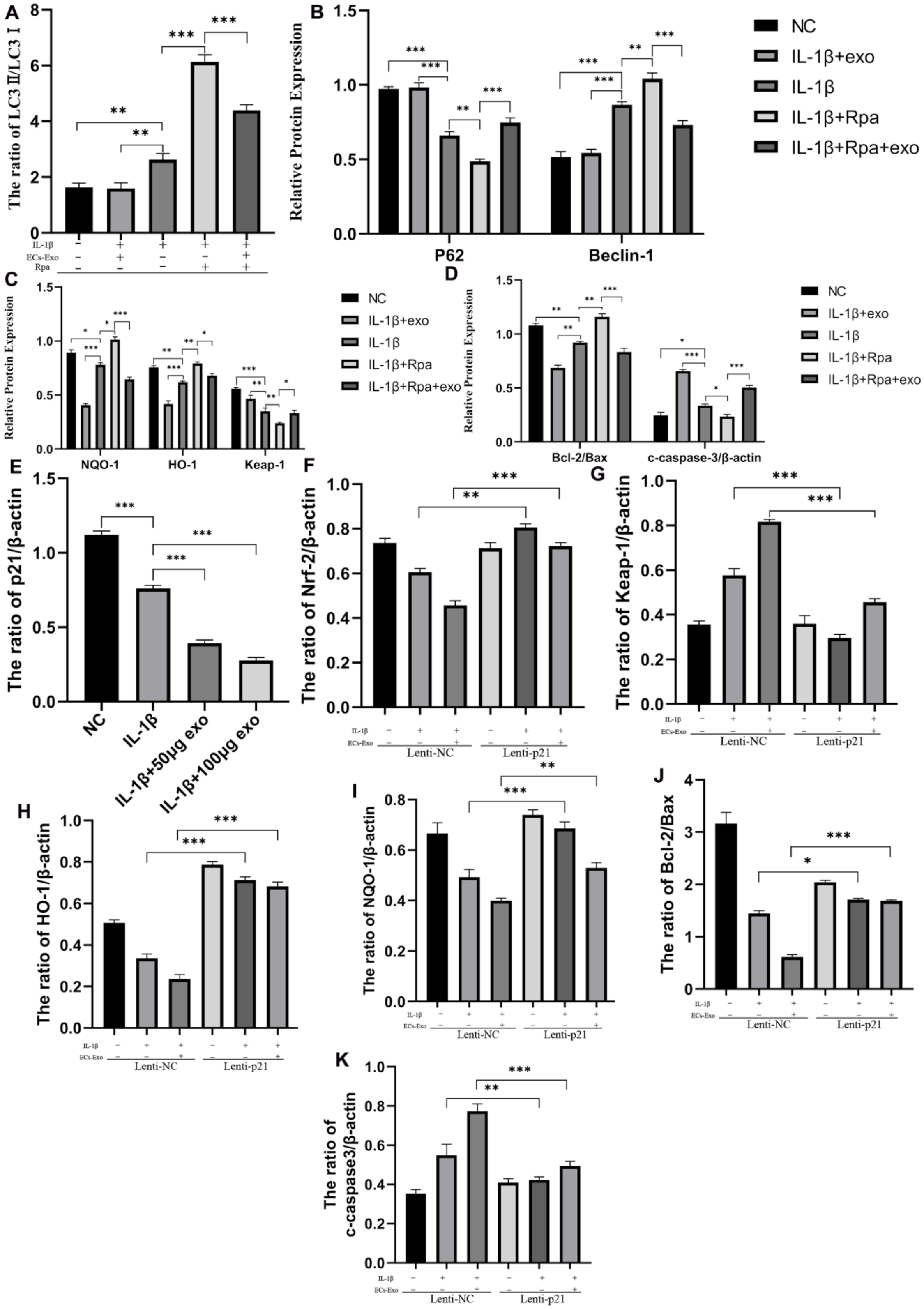

Supplementary Figure 2. (**A**) The ratio of LC3II/LC3I in ATDC5 cells under different treatments. (**B**) The ratio of autophagy marker proteins (p62 and Beclin-1) to β–actin in ATDC5 cells under different treatments. (**C**, **D**) The ratio of HO-1/β–actin, NQO-1/β–actin, Keap-1/β–actin, c-caspase3/β–actin and Bcl-2/Bax in ATDC5 cells under different treatments. (**E**) The ratio of p21/β–actin in ATDC5 cells in different groups. (**F**–**K**) The ratio of HO-1/β–actin, NQO-1/β–actin, Nrf-2/β–actin, Keap-1/β–actin, c-caspase3/β–actin and Bcl-2/Bax in ATDC5 cells under different treatments in the Lenti-NC group and Lenti-p21 group. ***p<0.001, **p<0.01, *p<0.05 (n = 3).
